# Supplementary material for: Resolving the full spectrum of human genome variation using Linked-Reads
Source: Genome Res. 2019 Apr;29(4):635–45. doi: 10.1101/gr.234443.118 (PMC6442396; doi:10.1101/gr.234443.118)
Supplement: Supplemental Material [file supp_gr.234443.118_Supplemental_longranger-2.2.zip › longranger-2.2.tar.bz2!/lr2.2-public/lib/stan/cmdstan/stan/doc/api/html/writer_8hpp_source.html]

Stan: src/stan/io/writer.hpp Source File


|  |  |
| --- | --- |
|  | Stan  2.5.0  probability, sampling & optimization |


- Main Page
- Related Pages
- Namespaces
- Classes
- Files

- File List
- File Members

All Classes Namespaces Files Functions Variables Typedefs Enumerations Enumerator Friends Macros Pages

- src
- stan
- io

writer.hpp

Go to the documentation of this file.

1 #ifndef STAN\_\_IO\_\_WRITER\_HPP

2 #define STAN\_\_IO\_\_WRITER\_HPP

3

4 #include <stdexcept>

5

6 #include <stan/math/matrix/meta/index\_type.hpp>

7 #include <stan/math/meta/index\_type.hpp>

8 #include <stan/prob/transform.hpp>

9

10

11 namespace stan {

12

13  namespace io {

14

27  template <typename T>

28  class writer {

29  private:

30  std::vector<T> data\_r\_;

31  std::vector<int> data\_i\_;

32  public:

33

34  typedef Eigen::Matrix<T,Eigen::Dynamic,Eigen::Dynamic> matrix\_t;

35  typedef Eigen::Matrix<T,Eigen::Dynamic,1> vector\_t;

36  typedef Eigen::Matrix<T,1,Eigen::Dynamic> row\_vector\_t;

37

38  typedef Eigen::Array<T,Eigen::Dynamic,1> array\_vec\_t;

39

44  const double CONSTRAINT\_TOLERANCE;

45

53  writer(std::vector<T>& data\_r,

54  std::vector<int>& data\_i)

55  : data\_r\_(data\_r),

56  data\_i\_(data\_i),

57  CONSTRAINT\_TOLERANCE(1E-8) {

58  data\_r\_.clear();

59  data\_i\_.clear();

60  }

61

65  ~writer() { }

66

73  std::vector<T>& data\_r() {

74  return data\_r\_;

75  }

76

77

84  std::vector<int>& data\_i() {

85  return data\_i\_;

86  }

87

93  void integer(int n) {

94  data\_i\_.push\_back(n);

95  }

96

104  void scalar\_unconstrain(T& y) {

105  data\_r\_.push\_back(y);

106  }

107

119  void scalar\_pos\_unconstrain(T& y) {

120  if (y < 0.0)

121  BOOST\_THROW\_EXCEPTION(std::runtime\_error ("y is negative"));

122  data\_r\_.push\_back(log(y));

123  }

124

136  void scalar\_lb\_unconstrain(double lb, T& y) {

137  data\_r\_.push\_back(stan::prob::lb\_free(y,lb));

138  }

139

150  void scalar\_ub\_unconstrain(double ub, T& y) {

151  data\_r\_.push\_back(stan::prob::ub\_free(y,ub));

152  }

153

166  void scalar\_lub\_unconstrain(double lb, double ub, T& y) {

167  data\_r\_.push\_back(stan::prob::lub\_free(y,lb,ub));

168  }

169

180  void corr\_unconstrain(T& y) {

181  data\_r\_.push\_back(stan::prob::corr\_free(y));

182  }

183

195  void prob\_unconstrain(T& y) {

196  data\_r\_.push\_back(stan::prob::prob\_free(y));

197  }

198

214  void ordered\_unconstrain(vector\_t& y) {

215  typedef typename stan::math::index\_type<vector\_t>::type idx\_t;

216  if (y.size() == 0) return;

217  stan::math::check\_ordered("stan::io::ordered\_unconstrain(%1%)",

218  y, "Vector");

219  data\_r\_.push\_back(y[0]);

220  for (idx\_t i = 1; i < y.size(); ++i) {

221  data\_r\_.push\_back(log(y[i] - y[i-1]));

222  }

223  }

224

241  void positive\_ordered\_unconstrain(vector\_t& y) {

242  typedef typename stan::math::index\_type<vector\_t>::type idx\_t;

243

244  // reimplements pos\_ordered\_free in prob to avoid malloc

245  if (y.size() == 0) return;

246  stan::math::check\_positive\_ordered("stan::io::positive\_ordered\_unconstrain(%1%)",

247  y, "Vector", (double\*)0);

248  data\_r\_.push\_back(log(y[0]));

249  for (idx\_t i = 1; i < y.size(); ++i) {

250  data\_r\_.push\_back(log(y[i] - y[i-1]));

251  }

252  }

253

254

260  void vector\_unconstrain(const vector\_t& y) {

261  typedef typename stan::math::index\_type<vector\_t>::type idx\_t;

262  for (idx\_t i = 0; i < y.size(); ++i)

263  data\_r\_.push\_back(y[i]);

264  }

265

271  void row\_vector\_unconstrain(const vector\_t& y) {

272  typedef typename stan::math::index\_type<vector\_t>::type idx\_t;

273  for (idx\_t i = 0; i < y.size(); ++i)

274  data\_r\_.push\_back(y[i]);

275  }

276

282  void matrix\_unconstrain(const matrix\_t& y) {

283  typedef typename stan::math::index\_type<matrix\_t>::type idx\_t;

284  for (idx\_t j = 0; j < y.cols(); ++j)

285  for (idx\_t i = 0; i < y.rows(); ++i)

286  data\_r\_.push\_back(y(i,j));

287  }

288

289  void vector\_lb\_unconstrain(double lb, vector\_t& y) {

290  typedef typename stan::math::index\_type<vector\_t>::type idx\_t;

291  for (idx\_t i = 0; i < y.size(); ++i)

292  scalar\_lb\_unconstrain(lb,y(i));

293  }

294  void row\_vector\_lb\_unconstrain(double lb, row\_vector\_t& y) {

295  typedef typename stan::math::index\_type<row\_vector\_t>::type idx\_t;

296  for (idx\_t i = 0; i < y.size(); ++i)

297  scalar\_lb\_unconstrain(lb,y(i));

298  }

299  void matrix\_lb\_unconstrain(double lb, matrix\_t& y) {

300  typedef typename stan::math::index\_type<matrix\_t>::type idx\_t;

301  for (idx\_t j = 0; j < y.cols(); ++j)

302  for (idx\_t i = 0; i < y.rows(); ++i)

303  scalar\_lb\_unconstrain(lb,y(i,j));

304  }

305

306  void vector\_ub\_unconstrain(double ub, vector\_t& y) {

307  typedef typename stan::math::index\_type<vector\_t>::type idx\_t;

308  for (idx\_t i = 0; i < y.size(); ++i)

309  scalar\_ub\_unconstrain(ub,y(i));

310  }

311  void row\_vector\_ub\_unconstrain(double ub, row\_vector\_t& y) {

312  typedef typename stan::math::index\_type<row\_vector\_t>::type idx\_t;

313  for (idx\_t i = 0; i < y.size(); ++i)

314  scalar\_ub\_unconstrain(ub,y(i));

315  }

316  void matrix\_ub\_unconstrain(double ub, matrix\_t& y) {

317  typedef typename stan::math::index\_type<matrix\_t>::type idx\_t;

318  for (idx\_t j = 0; j < y.cols(); ++j)

319  for (idx\_t i = 0; i < y.rows(); ++i)

320  scalar\_ub\_unconstrain(ub,y(i,j));

321  }

322

323

324  void vector\_lub\_unconstrain(double lb, double ub, vector\_t& y) {

325  typedef typename stan::math::index\_type<vector\_t>::type idx\_t;

326  for (idx\_t i = 0; i < y.size(); ++i)

327  scalar\_lub\_unconstrain(lb,ub,y(i));

328  }

329  void row\_vector\_lub\_unconstrain(double lb, double ub, row\_vector\_t& y) {

330  typedef typename stan::math::index\_type<row\_vector\_t>::type idx\_t;

331  for (idx\_t i = 0; i < y.size(); ++i)

332  scalar\_lub\_unconstrain(lb,ub,y(i));

333  }

334  void matrix\_lub\_unconstrain(double lb, double ub, matrix\_t& y) {

335  typedef typename stan::math::index\_type<matrix\_t>::type idx\_t;

336  for (idx\_t j = 0; j < y.cols(); ++j)

337  for (idx\_t i = 0; i < y.rows(); ++i)

338  scalar\_lub\_unconstrain(lb,ub,y(i,j));

339  }

340

341

342

358  void unit\_vector\_unconstrain(vector\_t& y) {

359  stan::math::check\_unit\_vector("stan::io::unit\_vector\_unconstrain(%1%)",

360  y, "Vector", (double\*)0);

361  typedef typename stan::math::index\_type<vector\_t>::type idx\_t;

362  vector\_t uy = stan::prob::unit\_vector\_free(y);

363  for (idx\_t i = 0; i < uy.size(); ++i)

364  data\_r\_.push\_back(uy[i]);

365  }

366

367

382  void simplex\_unconstrain(vector\_t& y) {

383  typedef typename stan::math::index\_type<vector\_t>::type idx\_t;

384

385  stan::math::check\_simplex("stan::io::simplex\_unconstrain(%1%)",

386  y, "Vector",

387  (double\*)0);

388  vector\_t uy = stan::prob::simplex\_free(y);

389  for (idx\_t i = 0; i < uy.size(); ++i)

390  data\_r\_.push\_back(uy[i]);

391  }

392

404  void cholesky\_factor\_unconstrain(matrix\_t& y) {

405  typedef typename stan::math::index\_type<matrix\_t>::type idx\_t;

406

407  // FIXME: optimize by unrolling cholesky\_factor\_free

408  Eigen::Matrix<T,Eigen::Dynamic,1> y\_free

409  = stan::prob::cholesky\_factor\_free(y);

410  for (idx\_t i = 0; i < y\_free.size(); ++i)

411  data\_r\_.push\_back(y\_free[i]);

412  }

413

414

426  void cholesky\_corr\_unconstrain(matrix\_t& y) {

427  typedef typename stan::math::index\_type<matrix\_t>::type idx\_t;

428

429  // FIXME: optimize by unrolling cholesky\_factor\_free

430  Eigen::Matrix<T,Eigen::Dynamic,1> y\_free

431  = stan::prob::cholesky\_corr\_free(y);

432  for (idx\_t i = 0; i < y\_free.size(); ++i)

433  data\_r\_.push\_back(y\_free[i]);

434  }

435

436

448  void cov\_matrix\_unconstrain(matrix\_t& y) {

449  typedef typename stan::math::index\_type<matrix\_t>::type idx\_t;

450  idx\_t k = y.rows();

451  if (k == 0 || y.cols() != k)

452  BOOST\_THROW\_EXCEPTION(

453  std::runtime\_error("y must have elements and"

454  " y must be a square matrix"));

455  idx\_t k\_choose\_2 = (k \* (k-1)) / 2;

456  array\_vec\_t cpcs(k\_choose\_2);

457  array\_vec\_t sds(k);

458  bool successful = stan::prob::factor\_cov\_matrix(y,cpcs,sds);

459  if (!successful)

460  BOOST\_THROW\_EXCEPTION(

461  std::runtime\_error ("factor\_cov\_matrix failed"));

462  for (idx\_t i = 0; i < k\_choose\_2; ++i)

463  data\_r\_.push\_back(cpcs[i]);

464  for (idx\_t i = 0; i < k; ++i)

465  data\_r\_.push\_back(sds[i]);

466  }

467

483  void corr\_matrix\_unconstrain(matrix\_t& y) {

484  typedef typename stan::math::index\_type<matrix\_t>::type idx\_t;

485

486  stan::math::check\_corr\_matrix("stan::io::corr\_matrix\_unconstrain(%1%)",

487  y, "Matrix",

488  (double\*)0);

489  idx\_t k = y.rows();

490  idx\_t k\_choose\_2 = (k \* (k-1)) / 2;

491  array\_vec\_t cpcs(k\_choose\_2);

492  array\_vec\_t sds(k);

493  bool successful = stan::prob::factor\_cov\_matrix(y,cpcs,sds);

494  if (!successful)

495  BOOST\_THROW\_EXCEPTION(

496  std::runtime\_error ("y cannot be factorized by factor\_cov\_matrix"));

497  for (idx\_t i = 0; i < k; ++i) {

498  // sds on log scale unconstrained

499  if (fabs(sds[i] - 0.0) >= CONSTRAINT\_TOLERANCE)

500  BOOST\_THROW\_EXCEPTION(

501  std::runtime\_error ("sds on log scale are unconstrained"));

502  }

503  for (idx\_t i = 0; i < k\_choose\_2; ++i)

504  data\_r\_.push\_back(cpcs[i]);

505  }

506

507  };

508  }

509

510 }

511

512 #endif

stan::io::writer::corr\_matrix\_unconstrain

void corr\_matrix\_unconstrain(matrix\_t &y)

Writes the unconstrained correlation matrix corresponding to the specified constrained correlation ma...

**Definition:** writer.hpp:483

stan::io::writer::row\_vector\_ub\_unconstrain

void row\_vector\_ub\_unconstrain(double ub, row\_vector\_t &y)

**Definition:** writer.hpp:311

stan::agrad::fabs

fvar< T > fabs(const fvar< T > &x)

**Definition:** fabs.hpp:18

stan::math::check\_ordered

bool check\_ordered(const char \*function, const Eigen::Matrix< T\_y, Eigen::Dynamic, 1 > &y, const char \*name, T\_result \*result)

Return true if the specified vector is sorted into increasing order.

**Definition:** check\_ordered.hpp:29

stan::io::writer::matrix\_unconstrain

void matrix\_unconstrain(const matrix\_t &y)

Write the specified unconstrained matrix.

**Definition:** writer.hpp:282

stan::prob::simplex\_free

Eigen::Matrix< T, Eigen::Dynamic, 1 > simplex\_free(const Eigen::Matrix< T, Eigen::Dynamic, 1 > &x)

Return an unconstrained vector that when transformed produces the specified simplex.

**Definition:** transform.hpp:1188

stan::io::writer::integer

void integer(int n)

Write the specified integer to the sequence of integer values.

**Definition:** writer.hpp:93

stan::io::writer::data\_r

std::vector< T > & data\_r()

Return a reference to the underlying vector of real values that have been written.

**Definition:** writer.hpp:73

stan::io::writer::array\_vec\_t

Eigen::Array< T, Eigen::Dynamic, 1 > array\_vec\_t

**Definition:** writer.hpp:38

stan::prob::prob\_free

T prob\_free(const T y)

Return the free scalar that when transformed to a probability produces the specified scalar...

**Definition:** transform.hpp:927

stan::prob::ub\_free

boost::math::tools::promote\_args< T, TU >::type ub\_free(const T y, const TU ub)

Return the free scalar that corresponds to the specified upper-bounded value with respect to the spec...

**Definition:** transform.hpp:668

stan::math::check\_simplex

bool check\_simplex(const char \*function, const Eigen::Matrix< T\_prob, Eigen::Dynamic, 1 > &theta, const char \*name, T\_result \*result)

Return true if the specified vector is simplex.

**Definition:** check\_simplex.hpp:32

stan::io::writer::scalar\_lub\_unconstrain

void scalar\_lub\_unconstrain(double lb, double ub, T &y)

Write the unconstrained value corresponding to the specified value with the specified bounds...

**Definition:** writer.hpp:166

stan::io::writer::cholesky\_factor\_unconstrain

void cholesky\_factor\_unconstrain(matrix\_t &y)

Writes the unconstrained Cholesky factor corresponding to the specified constrained matrix...

**Definition:** writer.hpp:404

stan::io::writer::positive\_ordered\_unconstrain

void positive\_ordered\_unconstrain(vector\_t &y)

Write the unconstrained vector that corresponds to the specified postiive ascendingly ordered vector...

**Definition:** writer.hpp:241

stan::io::writer::unit\_vector\_unconstrain

void unit\_vector\_unconstrain(vector\_t &y)

Write the unconstrained vector corresponding to the specified unit\_vector value.

**Definition:** writer.hpp:358

stan::io::writer::scalar\_lb\_unconstrain

void scalar\_lb\_unconstrain(double lb, T &y)

Return the unconstrained version of the specified input, which is constrained to be above the specifi...

**Definition:** writer.hpp:136

stan::math::check\_unit\_vector

bool check\_unit\_vector(const char \*function, const Eigen::Matrix< T\_prob, Eigen::Dynamic, 1 > &theta, const char \*name, T\_result \*result)

Return true if the specified vector is unit vector.

**Definition:** check\_unit\_vector.hpp:27

stan::io::writer::scalar\_unconstrain

void scalar\_unconstrain(T &y)

Write the unconstrained value corresponding to the specified scalar.

**Definition:** writer.hpp:104

stan::io::writer::row\_vector\_unconstrain

void row\_vector\_unconstrain(const vector\_t &y)

Write the specified unconstrained vector.

**Definition:** writer.hpp:271

stan::io::writer::simplex\_unconstrain

void simplex\_unconstrain(vector\_t &y)

Write the unconstrained vector corresponding to the specified simplex value.

**Definition:** writer.hpp:382

stan::math::index\_type

Primary template class for the metaprogram to compute the index type of a container.

**Definition:** index\_type.hpp:21

stan::prob::corr\_free

T corr\_free(const T y)

Return the unconstrained scalar that when transformed to a valid correlation produces the specified v...

**Definition:** transform.hpp:995

stan::io::writer::row\_vector\_lub\_unconstrain

void row\_vector\_lub\_unconstrain(double lb, double ub, row\_vector\_t &y)

**Definition:** writer.hpp:329

stan::io::writer::vector\_ub\_unconstrain

void vector\_ub\_unconstrain(double ub, vector\_t &y)

**Definition:** writer.hpp:306

stan::prob::unit\_vector\_free

Eigen::Matrix< T, Eigen::Dynamic, 1 > unit\_vector\_free(const Eigen::Matrix< T, Eigen::Dynamic, 1 > &x)

**Definition:** transform.hpp:1067

stan::io::writer::matrix\_lub\_unconstrain

void matrix\_lub\_unconstrain(double lb, double ub, matrix\_t &y)

**Definition:** writer.hpp:334

stan::io::writer::vector\_lb\_unconstrain

void vector\_lb\_unconstrain(double lb, vector\_t &y)

**Definition:** writer.hpp:289

stan::io::writer::cholesky\_corr\_unconstrain

void cholesky\_corr\_unconstrain(matrix\_t &y)

Writes the unconstrained Cholesky factor for a correlation matrix corresponding to the specified cons...

**Definition:** writer.hpp:426

stan::prob::cholesky\_corr\_free

Eigen::Matrix< T, Eigen::Dynamic, 1 > cholesky\_corr\_free(const Eigen::Matrix< T, Eigen::Dynamic, Eigen::Dynamic > &x)

**Definition:** transform.hpp:1592

index\_type.hpp

stan::prob::lub\_free

boost::math::tools::promote\_args< T, TL, TU >::type lub\_free(const T y, TL lb, TU ub)

Return the unconstrained scalar that transforms to the specified lower- and upper-bounded scalar give...

**Definition:** transform.hpp:845

stan::io::writer::cov\_matrix\_unconstrain

void cov\_matrix\_unconstrain(matrix\_t &y)

Writes the unconstrained covariance matrix corresponding to the specified constrained correlation mat...

**Definition:** writer.hpp:448

stan::math::check\_corr\_matrix

bool check\_corr\_matrix(const char \*function, const Eigen::Matrix< T\_y, Eigen::Dynamic, Eigen::Dynamic > &y, const char \*name, T\_result \*result)

Return true if the specified matrix is a valid correlation matrix.

**Definition:** check\_corr\_matrix.hpp:38

stan::io::writer::vector\_lub\_unconstrain

void vector\_lub\_unconstrain(double lb, double ub, vector\_t &y)

**Definition:** writer.hpp:324

stan::io::writer::matrix\_ub\_unconstrain

void matrix\_ub\_unconstrain(double ub, matrix\_t &y)

**Definition:** writer.hpp:316

stan::io::writer::matrix\_t

Eigen::Matrix< T, Eigen::Dynamic, Eigen::Dynamic > matrix\_t

**Definition:** writer.hpp:34

stan::prob::cholesky\_factor\_free

Eigen::Matrix< T, Eigen::Dynamic, 1 > cholesky\_factor\_free(const Eigen::Matrix< T, Eigen::Dynamic, Eigen::Dynamic > &y)

Return the unconstrained vector of parameters correspdonding to the specified Cholesky factor...

**Definition:** transform.hpp:1485

stan::io::writer::matrix\_lb\_unconstrain

void matrix\_lb\_unconstrain(double lb, matrix\_t &y)

**Definition:** writer.hpp:299

transform.hpp

stan::io::writer::row\_vector\_t

Eigen::Matrix< T, 1, Eigen::Dynamic > row\_vector\_t

**Definition:** writer.hpp:36

stan::math::E

const double E

The base of the natural logarithm, .

**Definition:** constants.hpp:14

stan::io::writer::vector\_t

Eigen::Matrix< T, Eigen::Dynamic, 1 > vector\_t

**Definition:** writer.hpp:35

stan::math::check\_positive\_ordered

bool check\_positive\_ordered(const char \*function, const Eigen::Matrix< T\_y, Eigen::Dynamic, 1 > &y, const char \*name, T\_result \*result)

Return true if the specified vector contains only non-negative values and is sorted into increasing o...

**Definition:** check\_positive\_ordered.hpp:29

stan::prob::factor\_cov\_matrix

bool factor\_cov\_matrix(const Eigen::Matrix< T, Eigen::Dynamic, Eigen::Dynamic > &Sigma, Eigen::Array< T, Eigen::Dynamic, 1 > &CPCs, Eigen::Array< T, Eigen::Dynamic, 1 > &sds)

This function is intended to make starting values, given a covariance matrix Sigma.

**Definition:** transform.hpp:88

stan::io::writer::data\_i

std::vector< int > & data\_i()

Return a reference to the underlying vector of integer values that have been written.

**Definition:** writer.hpp:84

stan::io::writer::scalar\_pos\_unconstrain

void scalar\_pos\_unconstrain(T &y)

Write the unconstrained value corresponding to the specified positive-constrained scalar...

**Definition:** writer.hpp:119

stan::io::writer::~writer

~writer()

Destroy this writer.

**Definition:** writer.hpp:65

stan::io::writer::vector\_unconstrain

void vector\_unconstrain(const vector\_t &y)

Write the specified unconstrained vector.

**Definition:** writer.hpp:260

stan::agrad::log

fvar< T > log(const fvar< T > &x)

**Definition:** log.hpp:15

index\_type.hpp

stan::io::writer::prob\_unconstrain

void prob\_unconstrain(T &y)

Write the unconstrained value corresponding to the specified probability value.

**Definition:** writer.hpp:195

stan::io::writer::corr\_unconstrain

void corr\_unconstrain(T &y)

Write the unconstrained value corresponding to the specified correlation-constrained variable...

**Definition:** writer.hpp:180

stan::io::writer::ordered\_unconstrain

void ordered\_unconstrain(vector\_t &y)

Write the unconstrained vector that corresponds to the specified ascendingly ordered vector...

**Definition:** writer.hpp:214

stan::io::writer::row\_vector\_lb\_unconstrain

void row\_vector\_lb\_unconstrain(double lb, row\_vector\_t &y)

**Definition:** writer.hpp:294

stan::io::writer::writer

writer(std::vector< T > &data\_r, std::vector< int > &data\_i)

Construct a writer that writes to the specified scalar and integer vectors.

**Definition:** writer.hpp:53

stan::io::writer::scalar\_ub\_unconstrain

void scalar\_ub\_unconstrain(double ub, T &y)

Write the unconstrained value corresponding to the specified lower-bounded value. ...

**Definition:** writer.hpp:150

stan::io::writer

A stream-based writer for integer, scalar, vector, matrix and array data types, which transforms from...

**Definition:** writer.hpp:28

stan::prob::lb\_free

boost::math::tools::promote\_args< T, TL >::type lb\_free(const T y, const TL lb)

Return the unconstrained value that produces the specified lower-bound constrained value...

**Definition:** transform.hpp:570

stan::io::writer::CONSTRAINT\_TOLERANCE

const double CONSTRAINT\_TOLERANCE

This is the tolerance for checking arithmetic bounds in rank and in simplexes.

**Definition:** writer.hpp:44


---

[ Stan Home Page ]

*© 2011–2014,
Stan Development Team.*
